# Rheumatoid arthritis developing after mogamulizumab treatment in Sézary syndrome

**DOI:** 10.1016/j.ero.2025.12.011

**Published:** 2026-01-03

**Authors:** Nozomi Nishimura, Mirei Shirakashi, Eiji Kiyohara, Akio Morinobu, Kosaku Murakami

**Affiliations:** 1 Department of Rheumatology and Clinical Immunology, Kyoto University Graduate School of Medicine, Kyoto, Japan; 2 Department of Dermatology, Graduate School of Medicine, The University of Osaka, Osaka, Japan; 3 Division of Clinical Immunology and Cancer Immunotherapy, Center for Cancer Immunotherapy and Immunobiology, Kyoto University Graduate School of Medicine, Kyoto, Japan

Dear Editor,

Mogamulizumab (MOG), a monoclonal antibody targeting CCR4, is approved for CCR4^+^ cutaneous T cell lymphomas, including Sézary syndrome [1]. In addition to its antitumor effects via antibody-dependent cellular cytotoxicity, MOG depletes CCR4^+^ regulatory T cells (Tregs) and other immune subsets, potentially disrupting immune tolerance [1]. Inflammatory arthritis associated with MOG is rare, with only a single prior case documented [2]. We present a rapidly progressive, erosive seronegative arthritis with accompanying mass cytometry (CyTOF) data.

A 74-year-old woman with Sézary syndrome (stage 4A1) refractory to bexarotene began MOG therapy in August of year X-1. She had no history of autoimmune disease. Autoimmune alopecia developed shortly after MOG initiation. Three months later, she presented with morning stiffness and symmetric polyarthritis. Physical examination showed swelling and tenderness of the bilateral metacarpophalangeal and proximal interphalangeal joints. There was no clinical evidence of tenosynovitis or enthesitis. At her first rheumatology evaluation in January of year X, serologic testing was negative for rheumatoid factor and anti-cyclic citrullinated peptide CCP antibodies. C-reactive protein (CRP) was 0.3 mg/dL (normal <0.04 mg/dL), and her Clinical Disease Activity Index (CDAI) was 12. Despite moderate disease activity, radiographs obtained at the same visit revealed multiple bone erosions and joint space narrowing in the proximal interphalangeal, metacarpophalangeal, and wrist joints (Fig, A). Nonsteroidal anti-inflammatory drugs were initiated at the patient’s request. However, symptoms worsened, and by 6 months, the CDAI was 33, and the C-reactive protein was 0.77 mg/dL. She fulfilled the 2010 American College of Rheumatology/European Alliance of Associations for Rheumatology classification criteria for rheumatoid arthritis (RA) [3]. At that time, the underlying lymphoma was worsening, and methotrexate (MTX) at 10 mg/wk was added to MOG, which also partially improved arthritis. However, MTX was discontinued after 2 months because of an inadequate tumour response and was replaced by etoposide. MOG and etoposide were eventually discontinued because of pleural effusion of unknown aetiology; however, arthritis persisted for more than one year after MOG discontinuation.

**Figure A:**
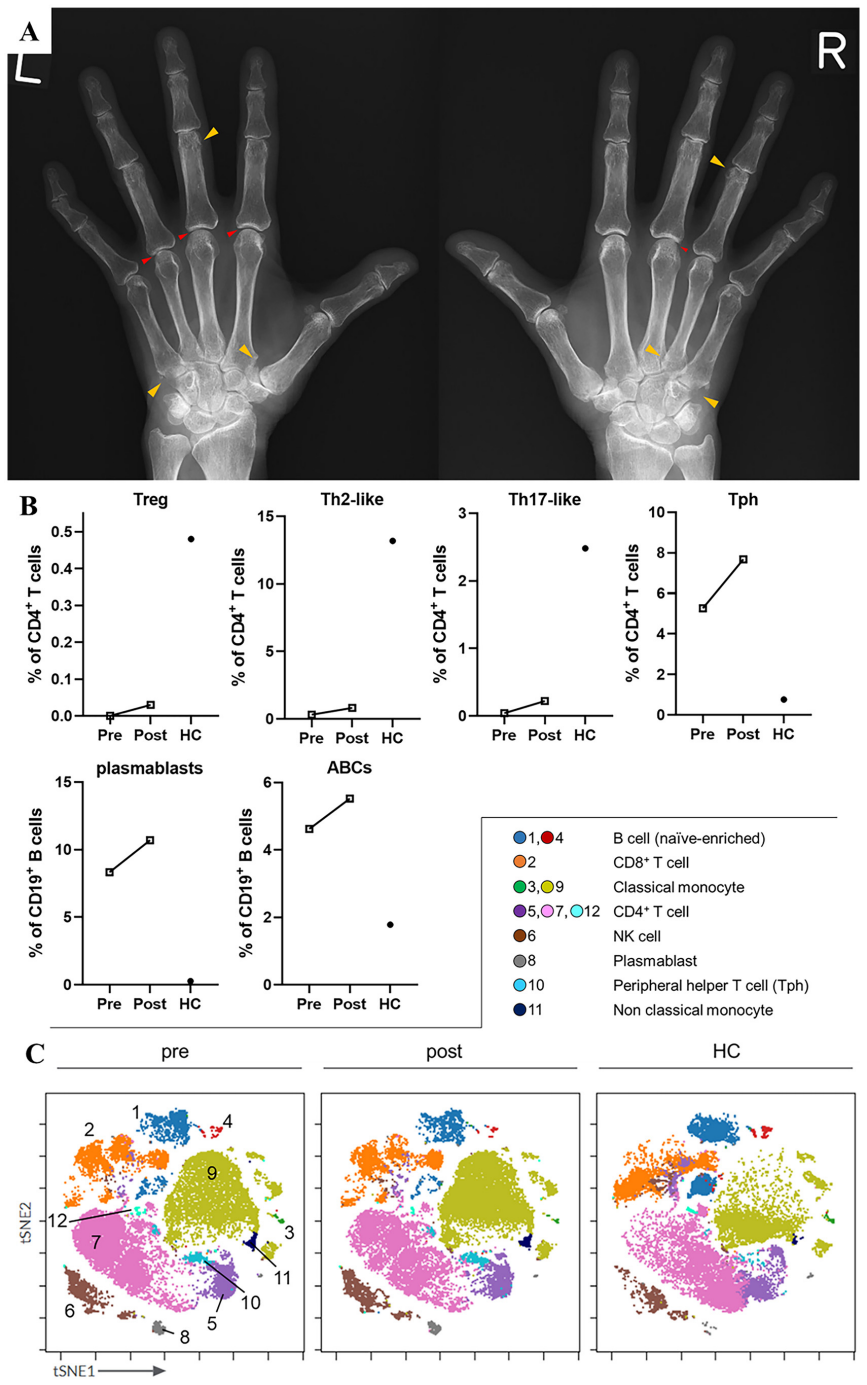
Radiologic and mass cytometry findings of mogamulizumab-induced inflammatory arthritis. A, Hand radiographs at initial evaluation showing multiple bone erosions (yellow) and joint space narrowing (red). B, CyTOF analysis of peripheral blood showing frequencies of Tregs, Th2-like, Th17-like, Tph, plasmablasts, and ABCs before (pre) and after (post) methotrexate treatment, and in a healthy control. Lines connect paired values from the same patient. ‘-like’ indicates phenotypic assignment by surface markers. C, viSNE maps of live CD45⁺ singlets (CD66b⁺ excluded) clustered into 12 FlowSOM metaclusters. Phenotypic labels are based on surface-marker expression (see Supplementary Table). ABC, aged-associated B cell; CyTOF, cytometry by time of flight; HC, healthy control; Tph, peripheral helper T cell; Treg, regulatory T cell; viSNE, visualization of t-distributed stochastic neighbor embedding; FlowSOM, flow self-organizing map.

MOG depletes CCR4^+^ tumour cells and secondarily removes CCR4^+^ immune subsets, not only resulting in enhanced antitumor immunity but also disrupting immune tolerance with a shift towards Th1 dominance. A prior CyTOF study in solid tumours similarly showed profound Treg depletion and increased activated CD4^+^ T cells after MOG administration, with little change in B cell subsets [4]. In our case, mass cytometry revealed marked depletion of Tregs, Th2, and Th17 cells in peripheral blood before and after MTX treatment (Fig, B,C). Additionally, the proportions of peripheral helper T cells (Tph), plasmablasts, and age-associated B cells (ABCs) were already elevated compared with those in a matched healthy control before MTX treatment and increased further after MTX treatment despite clinical improvement of arthritis. Importantly, MTX generally suppresses B cell activation in RA patients. However, in our case, these subsets paradoxically expanded despite MTX administration, suggesting a sustained MOG-induced autoimmune response that was insufficiently controlled by MTX. A subset of plasmablasts has been identified as autoreactive B cells in RA [5]. Together with these plasmablasts, ABCs and Tph promote extrafollicular B cell activation and differentiation. Tregs play a critical role in suppressing such pathogenic B cell responses [6]. In our patient, MOG administration precipitated immune dysregulation—Treg depletion along with expansion of plasmablasts, ABCs, and Tph—allowing autoreactive B cell activation and likely contributing to the development and persistence of inflammatory arthritis, even after MOG discontinuation. A previous report suggested that MOG could trigger seronegative RA-like polyarthritis, possibly via Treg depletion [2]. Our case extends these observations by providing mechanistic insight, representing a rapidly progressive, destructive form of seronegative RA that developed during MOG therapy. Notably, arthritis did not resolve spontaneously after drug discontinuation, suggesting that the breakdown of the immune profile involved more than just Treg depletion.

## Funding

No specific funding was received from any public, commercial, or not-for-profit sector bodies to conduct the work described in this article.

## Patient consent for publication

Informed consent was obtained for the publication of this article.

## Ethics approval

Not applicable.

## Provenance and peer review

Not commissioned; externally peer revoewed.

## Data availability statement

The data underlying this article will be shared upon reasonable request from the corresponding authors.

## CRediT authorship contribution statement

**Nozomi Nishimura:** Writing – original draft, Visualization, Validation, Software, Resources, Project administration, Methodology, Investigation, Formal analysis, Data curation, Conceptualization. **Mirei Shirakashi:** Writing – review & editing, Validation, Supervision, Software, Resources, Project administration, Methodology, Funding acquisition, Data curation, Conceptualization. **Eiji Kiyohara:** Writing – review & editing, Supervision, Investigation. **Akio Morinobu:** Writing – review & editing, Supervision. **Kosaku Murakami:** Writing – review & editing, Supervision, Project administration, Investigation, Funding acquisition, Data curation.

## Competing interests

All authors declare they have no competing interests.

## Appendix Supplementary materials

Image, application 1
