# Supplementary material for: Rheumatoid arthritis developing after mogamulizumab treatment in Sézary syndrome
Source: EULAR Rheumatol Open. 2026 Jan 3;2(2):100110. doi: 10.1016/j.ero.2025.12.011 (PMC13425175; doi:10.1016/j.ero.2025.12.011)
Supplement: Supplementary file 1 [file mmc1.docx]

**Supplementary Table S1**

**Supplementary Table S1. Marker profiles of peripheral blood immune cell metaclusters (CyTOF/FlowSOM).** CyTOF® data were obtained from peripheral blood live CD45⁺ singlets, with neutrophils and eosinophils (CD66b⁺) excluded. Samples included the patient before and after methotrexate treatment and a healthy control. The data were analyzed by viSNE and grouped into 12 metaclusters using FlowSOM. The table lists the assigned cell-subset labels and representative surface-marker expression for each metacluster. Expression symbols indicate relative levels within the same panel: “+” = positive, “int” = intermediate, “−/low” = negative to low, “hi” = high, and “variable” = heterogeneous within the cluster.

| **Metacluster (MC)** | **Subset name** | **Marker expression of each cluster** |
| --- | --- | --- |
| MC1, MC4 | B cell (Naïve-enriched) | IgD^+^, CD21^+^, CD20^+^, CD27^-/low^ |
| MC2 | CD8⁺ T cell | CD3^+^, CD8^+^, CXCR3^+^, CD57^+^, CCR7^-/low^, PD-1^-/low^, HLA-DR^-/low^ |
| MC3, MC9 | Classical monocyte | CD14^+^, HLA-DR^+^, CX3CR1^int^, CD16^-/low^ |
| MC5 | CD4⁺ T (central-memory) | CD3^+^, CD4^+^, CD27^+^, CCR7^+^, CD45RO^+^, PD-1^-/low^, ICOS^-/low^, HLA-DR^-/low^ |
| MC6 | NK cell | CD56^+,^ CD3^-/low^, CD16^variable^ |
| MC7 | CD4⁺ T (central-memory, PD-1^int^ ICOS^int^) | CD3^+^, CD4^+^, CD27^+^, CCR7^+^, CD127^+^, CD45RO^+^, PD-1^int^, ICOS^int^, CXCR5^-/low^ |
| MC8 | Plasmablast | CD38^hi^, HLA-DR^+^, CD27^+^, CD19^+^, CD20^-/low^, IgD^-/low^ |
| MC10 | T peripheral helper (Tph) | CD3^+^, CD4^+^, PD-1^hi^, ICOS^hi^, CD27^+,^ CXCR5^-/low,^ CCR7^int^, HLA-DR^int^ |
| MC11 | Non-classical monocyte | CD16^hi^, CX3CR1^+^, HLA-DR^+^, CD14^-/low^ |
| MC12 | CD4⁺ T (CD56⁺, small cluster) | CD3^+^, CD4^+^, CD56^+^, CD27^+^, CCR7^+^, CD127^+^, CD45RA^+^, CD38^+^, PD-1^-/low^ |
